# Heparin oligosaccharides: inhibitors of the biological activity of bFGF on Caco-2 cells.

**DOI:** 10.1038/bjc.1997.3

**Published:** 1997

**Authors:** G. C. Jayson, J. T. Gallagher

**Affiliations:** Cancer Research Campaign Department of Medical Oncology, Paterson Institute, Manchester, UK.

## Abstract

A number of growth factors, including members of the fibroblast growth factor (FGF) family - hepatocyte growth factor, vascular endothelial growth factor and heparin-binding epidermal growth factor - are dependent on heparan sulphate (HS) for biological activity mediated through their high-affinity signal-transducing receptors. This obligate requirement for HS prompted the search for antagonists of HS function that could be used as anti-growth factor drugs for the treatment of cancer. Basic FGF (bFGF) was the focus of this study. Caco-2, a human colon carcinoma cell line, was adapted to growth in serum-free medium so that investigation of its growth factor requirements for growth and migration could be performed in defined conditions (Jayson GC, Evans GS, Pemberton PW, Lobley RW, Allen T 1994, Cancer Res, 54, 5718-5723). This cell line multiplied and moved in a dose-dependent manner in response to bFGF. Here, we show that the mitogenic response to bFGF is dependent on the presence of heparan sulphate. A library of heparin oligosaccharides with uniform composition but variable length was generated [general formula [IdoA(2S)-GlcNS(6S)n], and oligosaccharides of defined lengths were tested for their ability to inhibit the biological activity of bFGF. While intact heparin and heparin-derived fragments of 12 monosaccharide units did not affect bFGF-induced cell division or bFGF-induced cell migration, octasaccharides and decasaccharides potently inhibited the bFGF-induced growth and migration responses. In particular, octasaccharides completely inhibited these biological activities at 10 microg ml-, a clinically achievable and tolerable concentration. This study shows that the length of an oligosaccharide determines its ability to block the biological activity of bFGF. The observation that the biological activity of cell-surface heparan sulphate can be antagonized in this way in a human carcinoma cell line suggests that oligosaccharides should be investigated further as anti-growth factor agents for the treatment of cancer. In addition, the results suggest that the clinical evaluation of low-molecular weight heparin (LMWH) as an anti-cancer agent might benefit from subfractionation of the LMWH, to remove oligosaccharides of 12 or more residues.


British Journal of Cancer (1997) 75(1), 9–16
© 1997 Cancer Research Campaign

### Heparin oligosaccharides: inhibitors of the biological activity of bFGF on Caco-2 cells

**GC Jayson and JT Gallagher**

Cancer Research Campaign Department of Medical Oncology, Paterson Institute, Wilmslow Road, Withington, Manchester M20 6BX, UK

**Summary** A number of growth factors, including members of the fibroblast growth factor (FGF) family – hepatocyte growth factor, vascular endothelial growth factor and heparin-binding epidermal growth factor – are dependent on heparan sulphate (HS) for biological activity mediated through their high-affinity signal-transducing receptors. This obligate requirement for HS prompted the search for antagonists of HS function that could be used as anti-growth factor drugs for the treatment of cancer. Basic FGF (bFGF) was the focus of this study. Caco-2, a human colon carcinoma cell line, was adapted to growth in serum-free medium so that investigation of its growth factor requirements for growth and migration could be performed in defined conditions (Jayson GC, Evans GS, Pemberton PW, Lobley RW, Allen T 1994, *Cancer Res*, **54**, 5718–5723). This cell line multiplied and moved in a dose-dependent manner in response to bFGF. Here, we show that the mitogenic response to bFGF is dependent on the presence of heparan sulphate. A library of heparin oligosaccharides with uniform composition but variable length was generated {general formula $[IdoA(2S)\text{-}GlcNS(6S)]_n$}, and oligosaccharides of defined lengths were tested for their ability to inhibit the biological activity of bFGF. While intact heparin and heparin-derived fragments of 12 monosaccharide units did not affect bFGF-induced cell division or bFGF-induced cell migration, octasaccharides and decasaccharides potently inhibited the bFGF-induced growth and migration responses. In particular, octasaccharides completely inhibited these biological activities at 10 μg $ml^{-1}$, a clinically achievable and tolerable concentration. This study shows that the length of an oligosaccharide determines its ability to block the biological activity of bFGF. The observation that the biological activity of cell-surface heparan sulphate can be antagonized in this way in a human carcinoma cell line suggests that oligosaccharides should be investigated further as anti-growth factor agents for the treatment of cancer. In addition, the results suggest that the clinical evaluation of low-molecular weight heparin (LMWH) as an anti-cancer agent might benefit from subfractionation of the LMWH, to remove oligosaccharides of 12 or more residues.

**Keywords:** basic fibroblast growth factor; inhibition; oligosaccharide; heparan sulphate; low molecular weight heparin; heparin

The majority of cancer-related morbidity and mortality in this country is caused by the growth and metastasis of adenocarcinoma (Association of Cancer Physicians, 1994). New treatment strategies are required to improve the prognosis of patients bearing these cancers, and inhibition of growth factor activity has been identified as a key approach (Myers et al, 1992; Eisenburger et al, 1993). Since a number of growth factors that are involved in cancer cell growth and angiogenesis (Mustonen and Alitalo, 1995) are dependent on heparan sulphate, we have investigated the potential of heparan sulphate oligosaccharides, which represent partial growth factor binding sequences, as inhibitors of growth factor activity.

Heparan sulphate (HS), a glycosaminoglycan (GAG), is a sulphated polymer of disaccharides that consists of an alternate arrangement of a hexuronic acid [either glucuronic acid or iduronic acid (IdoA)] and *N*-acetylated- or *N*-sulphated-glucosamine. HS is of particular interest because it contains domains of highly and poorly sulphated oligosaccharides (Turnbull and Gallagher, 1991), and the amount of sulphation differs depending on the cell of origin. While fibroblasts and aortic endothelial cells tend to make poorly sulphated HS, hepatocytes produce highly sulphated HS that resembles heparin in parts (Lyon et al, 1994*a*). HS exists as heparan sulphate proteoglycans in which it is covalently bound to a protein core and is responsible for binding a broad spectrum of ligands, including matrix proteins, protease inhibitors and growth factors.

*Received 8 May 1996*
*Revised 8 July 1996*
*Accepted 12 July 1996*

*Correspondence to:* GC Jayson

Heparan sulphate is found on the surface of nearly all cells in the body and one of its functions is to act as a co-receptor for a number of growth factors and chemokines. An enlarging family of growth factors, including members of the fibroblast growth factor (FGF) family (Olwin et al, 1992) – vascular endothelial growth factor (Gitay-Goren et al, 1992), hepatocyte growth factor (M Lyon, JA Deakin and JT Gallagher, personal communication) and heparin-binding epidermal growth factor (Higashiyama et al, 1993) – show an obligate requirement for heparan sulphate in order to activate their high-affinity signal-transducing receptors.

The heparan sulphate co-receptor is necessary for bFGF to bind (Yayon et al, 1991) and activate (Olwin et al, 1992) its signal-transducing receptor. The interaction between HS and bFGF is specific, and a 14-saccharide HS sequence has been identified, containing an internal sequence of five IdoA(2S)-GlcNS disaccharide units, which bind bFGF with an affinity similar to intact HS. Oligosaccharides containing fewer copies of this disaccharide bound bFGF with a reduced affinity (Turnbull et al, 1992). When the biological activity of similar oligosaccharides was examined, there were data to suggest that oligosaccharides of eight or fewer saccharide residues were capable of binding bFGF but would not support the biological activity of the cytokine, whereas oligosaccharides of 10 or 12 saccharides both bound and activated bFGF in endothelial (Ishihara et al, 1993) and mesenchymal model systems (Walker et al, 1994). This suggested that short oligosaccharides were capable of acting as growth factor inhibitors. However, many of these studies were carried out in cell-free systems or on HS-denuded cells in which the oligosaccharide under investigation did not have to compete with cell-surface or extracellular matrix HS for the growth factor. Secondly, the composition of oligosaccharides of different lengths was not homogeneous and whether oligosaccharide length alone (rather than compositional variations) could determine the inhibitory potential of these molecules was unknown. To overcome these problems, oligosaccharides of general formula $[IdoA(2S)\text{-}GlcNS(6S)]_n$ were generated by chemical scission of bovine lung heparin, and their biological activity was tested. Each of these disaccharide units contains the sulphate groups that have been implicated in the binding and activation of bFGF, namely the *N*-sulphate on glucosamine and the 2-*O*-sulphate on iduronic acid. The 6-*O*-sulphate is not thought to be important to the interaction between HS and bFGF (Coltrini et al, 1994; Ishihara et al, 1994), although it has been implicated in the interaction between HS and the FGF receptor (Guimond et al, 1993).

The action of HS in the regulation of the growth response of carcinoma cells to bFGF has not been investigated to date. In view of the specificity of the interaction between bFGF and HS and the mandatory role of HS in bFGF signalling, the apparent HS-mediated activation of the growth factor is a potential target for therapeutic control. To investigate these possibilities, we adapted Caco-2, a human colon carcinoma cell line, to growth in serum-free, defined conditions. As this cell line responds to bFGF in a dose-dependent manner by increasing its rate of multiplication and motility (Jayson et al, 1994), we were able to test the relative potencies of heparin oligosaccharides of general formula $[IdoA(2S)\text{-}GlcNS(6S)]_n$ (where $n$ = 1–6) as inhibitors of the mitogenic and motogenic effects of bFGF.

### MATERIALS AND METHODS

### Cell culture

The adaptation of Caco-2 to growth in serum-free conditions and the materials and methods for measuring the mitogenic and motogenic response of Caco-2 to bFGF were outlined in detail in Jayson et al (1994).

Briefly, Caco-2 was adapted to growth in serum-free conditions [Dulbecco's modified Eagle medium (DMEM) containing pyruvate, glutamine, penicillin and streptomycin with 10 μg $ml^{-1}$ transferrin (Collaborative Biomedicals) (D+T)] by reducing the serum concentration at each passage so that the cells eventually grew in serum-free conditions and underwent differentiation on reaching confluence, in the same way as the parental cell line.

### Determination of the concentration of chlorate needed to prevent the formation of sulphated GAG

Caco-2 cells were taken at 80% confluence in a T75 flask (Costar) and incubated for 3 h in sulphate-free DMEM, containing between 0 and 5 mM sodium chlorate, with a 90% and 75% reduction in cysteine and methionine respectively, and no penicillin or streptomycin. The other constituents of D+T were otherwise the same. After 3 h, the cells were passaged into another T75 flask containing the same concentration of sodium chlorate, but also containing 10 μCi $ml^{-1}$ [$^3$H]glucosamine (NEN, DuPont) and 10 μCi $ml^{-1}$ $Na_2{}^{35}SO_4$ (NEN, DuPont). After 48 h, 1% (v/v) Triton X-100 (Sigma) and 10 mg pronase (*Streptomyces griseus*, Boehringer Mannheim) were added to liberate GAG. The sample was filtered (2 μm filter, Millipore), diluted twice (distilled water, v/v) and then loaded onto a Mono-Q anion exchange column in a fast protein liquid chromatographic (FPLC) apparatus (Pharmacia). The column was equilibrated with water and a linear gradient up to 2 M sodium chloride was used to elute the retained material. Fractions were collected and their radioactivity determined by scintillation counting.

### The mitogenic response of Caco-2 to bFGF is dependent on heparan sulphate

Caco-2 at 80% confluence, in 75-$cm^2$ flasks, were incubated for 3 h in 10 mM sodium chlorate in sulphate-free DMEM (Gibco) containing glutamine, pyruvate and transferrin. Penicillin and streptomycin were omitted as they contain sulphur, and cysteine and methionine were reduced to 10% and 25% of normal concentrations respectively, as they could also provide exogenous sulphate, which would overcome the effect of chlorate.

Cells were liberated as a single-cell suspension with trypsin that was subsequently neutralized with soybean trypsin inhibitor (Jayson et al, 1994). Approximately $10^5$ cells in 1 ml of chlorate-containing medium were pipetted into each well of a 24-well plate (Costar, flat-bottomed wells). For the first 24 h the cells were allowed to adhere in the chlorate-containing medium, so that there were an equal number of cells in each well.

After 24 h, the medium was changed and growth factors, glycosaminoglycans and 1 mM magnesium sulphate were added in various combinations (see Results) to the chlorate-containing medium. Growth factors and GAGs were used at a concentration of 10 ng $ml^{-1}$, and the cultures were incubated for 24 h. An aliquot of 1 μCi of [$^3$H]thymidine (NEN, Dupont) was then added to each well. Three hours later, the medium containing the radiolabel was aspirated, and the cells were fixed with ice-cold methanol (BDH) for 1 h. The cells were treated with at least three washes with ice-cold 5% trichloroacetic acid (TCA, Sigma) over 18 h to remove any thymidine that had not been incorporated into DNA. They were then washed in ethanol and dried in air. The DNA, containing the thymidine, was dissolved in 0.75 ml of 1 M sodium hydroxide at 37°C for 3 h, and the completion of the solubilization was determined by light microscopy. The alkali was neutralized with 0.75 ml of 1 M hydrochloric acid and 0.5 ml was mixed with scintillant, and the radioactivity counted on a scintillation counter (Packard Tricarb 4660, Canberra Packard). To standardize the findings, the mean radioactivity in the chlorate wells was defined as 100%. The *y*-axis shows the percentage increase in DNA synthesis in excess of that seen in the chlorate wells. The results are shown (Figure 2) as the mean of at least three experiments (± s.e.). A linear relationship between cell number and [$^3$H] thymidine uptake was established (data not shown).

### Analysis of bovine lung heparin (BLH)

A sample of 50 mg of BLH (Sigma) was dissolved in 1 ml of 50 mM Tris (Sigma), 50 mM sodium chloride (BDH), pH 7.8–8 and exhaustively digested with chondroitinase ABC (EC 4.2.2.4, Seikagaku, Tokyo, Japan) for 24 h at 37°C to remove any contamination with chondroitin or dermatan sulphate. The intact heparin

© Cancer Research Campaign 1997

was separated from digestion products by anion exchange chromatography using a 10 ml DEAE-Sepharose (Sigma) column and was loaded onto a 1 × 30 cm Sephadex G-50 superfine column (Pharmacia: fractionation range 500–10 000 MW), pre-equilibrated with 0.2 M ammonium bicarbonate (BDH) at 20 ml per hour. The elution position of the heparin was determined by the measurement of absorption (232 nm), and those fractions containing the heparin were pooled and exhaustively lyophilized. The heparin was dissolved in 5 ml of heparinase buffer [0.1 M sodium acetate (BDH), 0.1 M calcium acetate (BDH), 0.1 mg $ml^{-1}$ bovine serum albumin (Sigma), pH 7] and exhaustively digested with heparinase I, II and III (Seikagaku, Tokyo, Japan) at 37°C for 24 h. The degree of digestion was measured by following the absorption (232 nm) of fractions eluted by gel chromatography [Biogel P2, (Biorad, Hertfordshire, UK), 1 × 150 cm, 5 ml of 0.2 M ammonium bicarbonate per hour] and was shown to be greater than 95% (data not shown).

The fractions corresponding to the disaccharides were pooled and exhaustively lyophilized to remove ammonium bicarbonate. The disaccharides were dissolved in 1 ml of water and 10 µl were analysed by strong anion exchange high-performance liquid chromatography (HPLC) on a Propac PA1 analytical column (4 × 250 mm, Dionex, UK). The elution was followed at 232 nm.

### Preparation of oligosaccharides from bovine lung heparin (BLH)

A sample of 50 mg of BLH (Sigma) was treated as above to remove any contamination with chondroitin or dermatan sulphate.

The oligosaccharides derived from BLH were produced by the random chemical scission of intact heparin with nitrous acid, which cleaves heparin at *N*-sulphated disaccharides. The lyophilized heparin was dissolved in 1 ml of water and separated into five aliquots of 200 µl. Nitrous acid was prepared by mixing 0.5 ml of water containing 0.114 g of barium nitrite (Aldrich Chemical Co.) with 0.5 ml of 0.5 M sulphuric acid and taking the supernatant after (5 min) centrifugation at 13 000 ***g*** (Shively and Conrad, 1976). An aliquot of 50 µl of nitrous acid solution was added to each aliquot of heparin and the reaction was allowed to proceed for 5, 10, 15, 20 and 25 s respectively, before it was terminated by the addition of 0.3 ml of 2 M sodium carbonate. The five aliquots were mixed and the total volume was reduced to less than 1 ml by centrifugal evaporation (Uniscience, Univap). The oligosaccharides were separated on a 1.5 × 150 cm Biogel P10 column (Biorad) pre-equilibrated with 0.2 M ammonium bicarbonate, running at 8 ml per hour. Aliquots (2 ml) were collected and the absorption of each was measured at 210 nm using a spectrophotometer (Cecil 5501 series 5000 spectrophotometer, Cecil, Cambridge, UK) (Figure 3). Those aliquots containing the hexasaccharides (dp6), octasaccharides (dp8), decasaccharides (dp10) and dodecasaccharides (dp12) were taken and exhaustively lyophilized to remove the ammonium bicarbonate. These were dissolved in D+T (no transferrin), sterilized by filtration through 0.2 µm filters and used in the assays of migration and mitogenesis.

In some experiments, the biological activity of chondroitin sulphate oligosaccharides was investigated. Chondroitin sulphate (50 mg, Sigma) was dissolved in chondroitinase buffer (50 mM Tris, 50 mM sodium chloride, pH 7.8–8) and digested with chondroitinase ABC (*Proteus vulgaris*, EC 4.2.2.4, Seikagaku, Tokyo, Japan) for 2 h at 37°C. The digested material was then eluted through the same Biogel P10 column and the absorption (232 nm) of the eluant was determined. Hexasaccharides and octasaccharides were taken for exhaustive lyophilization and investigation in the biological assays.

### Mitogenic assay

Caco-2 were taken at confluence and liberated as a single-cell suspension with trypsin. The trypsin was neutralized with soybean trypsin inhibitor (Sigma) and the cells were resuspended in D+T, which contained 1 µg $ml^{-1}$ transferrin (Jayson et al, 1994). A sample of 100 µl containing $10^4$ cells was pipetted into each well of a 96-well flat-bottomed plate (Costar). After 24 h, the cells had adhered and the medium was changed to D+T, which did not contain transferrin. Where appropriate, bFGF (R&D Systems, USA) was added to give a final concentration of 10 ng $ml^{-1}$, and oligosaccharides were added to produce final concentrations of 1, 10 or 100 µg $ml^{-1}$. The cells were incubated for 4 days and then fixed in 2% glutaraldehyde (BDH)/Hanks' balanced salt solution (HBSS) (Gibco) (v/v) for 1 h at room temperature. The cells were washed and then stained with 0.1% crystal violet (w/v) in 50 mM sodium tetraborate buffer (pH 9) for 20 min. After washing in water, the stain was liberated with 100 µl of 10% acetic acid, and the absorption was measured using an Anthos Labtec (Austria) plate reader at 540 nm. The influence of the oligosaccharide on bFGF-induced mitogenesis was calculated by

$$[(A_{540} \text{ of cells in bFGF + oligosaccharide}/A_{540} \text{ of control cells}) - (A_{540} \text{ of cells in oligosaccharide}/A_{540} \text{ of control cells})] \times 100$$

A linear relationship existed between cell number and uptake of crystal violet stain (data not shown). The results are presented as the increase in cell number in the FGF/oligosaccharide-treated wells compared with that in the wells treated with oligosaccharide alone. The oligosaccharide concentrations were 0, 1, 10 and 100 µg $ml^{-1}$. The data are presented as the mean result of three experiments (± s.e.) in Figure 5A. The control experiments performed with chondroitin sulphate oligosaccharides are shown in Figure 5B.

### Migration assay

The migration assay involved the measurement of the percentage of cells that migrated across a polycarbonate membrane containing 12-µm pores in 24 h in response to bFGF in the presence or absence of oligosaccharides.

Caco-2 were taken at confluence, liberated as a single-cell suspension with trypsin that was then neutralized with soybean trypsin inhibitor, and the cells were resuspended in D+T (without transferrin) at $10^5$ cells $ml^{-1}$ (Jayson et al, 1994). A sample of 0.5 ml of cells was pipetted into a well, the base of which was a polycarbonate membrane containing 12-µm holes (Costar Transwells). Aliquots of 0.5 ml of D+T (without transferrin), containing bFGF at 100 ng $ml^{-1}$ and/or oligosaccharide at 0, 10 or 100 µm $ml^{-1}$ were added below the membrane. After 24 h, the media above and below the membrane were aspirated and 1 ml of 2% glutaraldehyde/HBSS was added above and below the membrane for at least 1 h at room temperature. The membranes were then washed with distilled water and stained with 5 µg $ml^{-1}$ Hoechst dye (Sigma) for 5 min at room temperature. After two further washes with distilled water, the membranes were excised, their orientation being maintained, and mounted in water on glass slides and examined under a Zeiss phase-contrast fluorescence microscope. At least 1000 cells were counted per membrane, and the percentage of cells that had

© Cancer Research Campaign 1997

**Figure 1** Effect of chlorate on [$^{35}$S]GAG formation. Anion exchange chromatographs showing the reduction in [$^3$H] glucosamine/[$^{35}$S]GAG peaks when Caco-2 cells were incubated in medium containing different concentrations of sodium chlorate. GAG were eluted from a mono-Q anion exchange column by a linear sodium chloride gradient (0–2 M). Left axis: solid line, tritium; fine dotted line, sulphur-$^{35}$. Right axis: linear dotted line, saline gradient migrated across the membrane was determined. The results shown in Figure 6 were calculated by the formula:

% of cells that migrated in excess of control =
[(% of cells migrating in bFGF + oligosaccharide) –
(% of cells migrating in oligosaccharide)]

Each experiment was repeated three times and the standard errors calculated. The results are shown in Figure 6.

### RESULTS

Figure 1 shows that chlorate was able to reduce the production of sulphated glycosaminoglycans in a dose-dependent manner. The graphs show that the glycosaminoglycan (GAG) peak, identified by its uptake of both [$^3$H]glucosamine and $^{35}$S, was reduced by >95% when the cells were incubated in 5 mM sodium chlorate.

**Figure 2** Effect of heparin and other sulphated GAG on the mitogenic activity of bFGF. Heparin is required for the mitogenic activity of bFGF. Caco-2 cells were incubated in 10 mM chlorate for 24 h, passaged into fresh medium containing FGF, 10 ng ml$^{-1}$ basic FGF; CS, 10 ng ml$^{-1}$ chondroitin sulphate; $SO_4$, 1 mM magnesium sulphate; EGF, 10 ng ml$^{-1}$ epidermal growth factor. The data represent the mean ± s.e. for at least three experiments

A concentration of 10 mM chlorate was selected for further experiments to guarantee a complete reduction in GAG synthesis.

Caco-2 cells were grown in defined conditions in chlorate-containing, low-sulphate medium with other additives as shown in the legend for Figure 2. The data suggest that heparan sulphate is required for the mitogenic effect of bFGF on Caco-2. The addition of heparin to chlorate-treated cells was associated with a minimal increase (6%) in DNA synthesis in comparison with the background level observed with chlorate-treated cells. bFGF alone caused a small stimulation of 20% in excess of control, despite the treatment of cells with chlorate. However, the addition of bFGF with heparin caused a 68% increase in DNA synthesis, which was significantly greater than the sum of bFGF and heparin when either was given alone, suggesting a strong synergy between the two and supporting the concept that bFGF is activated by heparin.

Chlorate inhibits the production of sulphated glycosaminoglycans by a competitive inhibition of the manufacture of the physiological sulphate donor, phosphoadenosine phosphosulphate (Baeuerle and Huttner, 1976; Rapraeger, 1991). However, this inhibition can be reversed by the exogenous addition of sulphate. Thus, treatment of the cells with sulphate, despite the presence of chlorate, was associated with a 30% increase in DNA synthesis, while incubation of cells in sulphate and bFGF was associated with a 90% increase in DNA synthesis. The difference between the sulphate/bFGF and sulphate data points is 60%, a figure that is similar to the difference between the heparin/bFGF (68% stimulation) and heparin (6% stimulation) data points. Therefore, these results imply that heparin in solution is equivalent to cell-surface HS in its ability to promote the activity of bFGF.

The ability of chondroitin sulphate (CS) to restore the mitogenic effect of bFGF was investigated. This showed that CS alone caused an insignificant increase in DNA synthesis, whereas CS with bFGF caused 11% stimulation. Since bFGF alone caused 20% stimulation, the addition of CS had no significant effect on the mitogenic effect of bFGF. This suggests that the mitogenic activity of bFGF in Caco-2 depends on heparan sulphate, rather than on other sulphated glycosaminoglycans.

In addition, we investigated the influence of heparin on the mitogenic effect of epidermal growth factor (EGF) in chlorate-treated

 © Cancer Research Campaign 1997

**Figure 3** The disaccharide composition of bovine lung heparin. Bovine lung heparin was completely depolymerized by scission with heparinases I, II and III. Disaccharides were extracted, washed onto a strong anion exchange column and desorbed by a linear saline gradient. Left axis, $A_{232}$, solid line; right axis, linear saline gradient, dotted line

**Figure 4** Isolation of heparin oligosaccharides. Bovine lung heparin was randomly cleaved with nitrous acid and the products were separated by Biogel P10 gel filtration chromatography. The $A_{210}$ of eluant corresponded to oligosaccharides of different sizes. dp6, hexasaccharides; dp8, octasaccharides; dp10, decasaccharides; dp12; dodecasaccharides. Salt, salts produced by the neutralization of nitrous acid with sodium carbonate cells. EGF is a non-heparin-binding growth factor and, therefore, heparin should not increase the mitogenic activity of EGF. The data show that EGF alone caused a 41% increase in DNA synthesis (less than bFGF and heparin), whereas EGF with heparin caused a 55% increase. This minor effect of heparin is likely to be caused by an independent action of the polysaccharide since, as indicated above, heparin does cause a small increase in DNA synthesis in the absence of exogenous growth factor.

### Compositional analysis of bovine lung heparin

Figure 3 shows the composition of bovine lung heparin after complete depolymerization with heparin lyases (I–III) and separation of the disaccharides on strong anion exchange HPLC. The results show that 85% of the disaccharides are the trisulphated disaccharide IdoA(2S)-GlcNS(6S) and that 95% of all disaccharides contain GlcNS and IdoA(2S), sulphated moieties implicated in the interaction between bFGF and heparan sulphate (Turnbull et al, 1992; Ishihara et al, 1994; Maccarana et al, 1993). These data suggest that oligosaccharides derived from bovine lung heparin will be quite homogeneous in composition and that the biological activity of oligosaccharides will result from differences in length rather than composition.

### Preparation of oligosaccharides from bovine lung heparin

Figure 4 shows the typical absorption (210 nm) profile of oligosaccharides, derived by chemical scission of bovine lung heparin, eluted by Biogel P10 gel filtration chromatography. The column was precalibrated with radiolabelled oligosaccharides, so that oligosaccharides of known length could be identified. The dp labels refer to the degree of polymerization, so that dp6 refers to hexasaccharides; dp8 to octasaccharides and dp12 to dodecasaccharides. Oligosaccharides were separated by taking the fraction containing the highest amount of an oligosaccharide and the fractions either side of the main fraction. These were exhaustively lyophilized until there was no further weight loss. Gradient-polyacrylamide gel electrophoresis (PAGE) analysis showed that oligosaccharides of a stated length contained at least 90% of that length of oligosaccharide (data not shown).

### The inhibition of bFGF-induced mitogenesis by oligosaccharides

Heparin oligosaccharides were added, with or without bFGF, at the concentrations shown on the $x$-axis of Figure 5A. The difference in cell number between wells that were treated with oligosaccharide and bFGF and oligosaccharide alone was plotted for each oligosaccharide at the concentration shown. The results show that intact heparin and dodecasaccharides do not inhibit bFGF-induced mitogenesis, even at 100 μg $ml^{-1}$. On the other hand, hexasaccharides, octasaccharides and decasaccharides inhibited the mitogenic activity of bFGF at 100 μg $ml^{-1}$. However, the octasaccharides also caused a significant reduction in mitogenesis at 10 μg $ml^{-1}$, whereas neither the hexasaccharides nor the decasaccharides did this. To investigate the specificity of the effect, the inhibitory potential of CS hexasaccharides and octasaccharides was investigated. Figure 5B shows that these largely had a statistically insignificant effect on bFGF-induced mitogenesis, suggesting that a specific class of GAG oligosaccharides was required to inhibit the biological activity of bFGF.

### The inhibition of bFGF-induced motility by oligosaccharides

Basic FGF causes a dose-dependent increase in migration (Jayson et al, 1994). This was demonstrated by counting cells that migrated across a transwell filter (pore size 12 μm) over a 24-h period. In the

© Cancer Research Campaign 1997

**Figure 5** The inhibitory activity of sulphated GAG oligosaccharides. (**A**) Effect of heparin oligosaccharides on bFGF-induced mitogenesis. (**B**) Effect of chondroitin sulphate oligosaccharides on bFGF-induced mitogenesis. ◆, hexasaccharides; ■, octasaccharides; ▲, decasaccharides; ×, dodecasaccharides; ●, intact heparin. Inset shows dose–response curve for bFGF-induced Caco-2 mitogenesis. * Significant reduction in the mitogenic activity of oligosaccharides ($P$<0.05; Student's two-tailed $t$-test)

**Figure 6** Effect of heparin-derived oligosaccharides on bFGF-induced cell motility. Caco-2 cells were pipetted above a membrane containing 12-µm holes and 100 ng ml$^{-1}$ bFGF with oligosaccharides of defined length and the concentrations shown on the $x$-axis were added below the membrane. Each experiment was repeated three times and the mean ± s.e. are shown. ◆, hexasaccharides; ■, octasaccharides; ▲, dodecasaccharides. The inset shows a typical dose–response curve for bFGF-induced Caco-2 migration through the membrane. * Significant reduction in the mitogenic activity of oligosaccharides ($P$<0.05; Student's two-tailed $t$-test)

present study, we have used the same assay to study the effects of oligosaccharide antagonists of bFGF-induced mitogenesis on the migration response of cells to this growth factor. The small inset in Figure 6 shows a typical dose migration curve for Caco-2 cells. Figure 6 shows that short oligosaccharides reduce the bFGF-induced motility of Caco-2 cells. Octasaccharides were again the most potent inhibitors of cell motility, as they abolished bFGF-induced cell migration at 10 µg ml$^{-1}$. Hexasaccharides caused an insignificant reduction in motility at 10 µg ml$^{-1}$ but eliminated bFGF-induced motility at 100µg ml$^{-1}$. Dodecasaccharides had no effect on cell motility at 10 µg ml$^{-1}$ but caused a slight reduction in cell motility at 100 µg ml$^{-1}$ that did not achieve statistical significance.

### DISCUSSION

The evidence that the mitogenic effect of bFGF is dependent on HS was derived from mesenchymal models. This is the first demonstration that the mitogenic effect of bFGF in an epithelial model of cancer is dependent on heparan sulphate (Figure 2). The data show that heparin, but not chondroitin sulphate, was able to restore completely the mitogenic effect of bFGF in cells denuded of functional heparan sulphate. However, the mitogenic effect of EGF, a non-heparin-binding growth factor, was only slightly affected by heparin. This suggests that there is a specific requirement by bFGF for heparan sulphate rather than an alternative glycosaminoglycan. This may be mediated either by a synergism between heparan sulphate receptors and FGF receptors or by an HS–bFGF–FGF receptor interaction. However, there are no convincing reports of heparin/heparan sulphate receptors in the literature. In addition, neither heparin alone nor heparin in the presence of EGF increased DNA synthesis over control levels (chlorate medium and EGF respectively), supporting the concept that heparin increases the mitogenic effect of bFGF by a modulation of the bFGF–FGF receptor system, rather than through a synergistic effect mediated through alternative cell-surface receptors.

The dependency of bFGF on heparan sulphate, of minimum length 12–14 saccharides, for its biological activity prompted the investigation of shorter heparan sulphate oligosaccharides that represented part of the active site sequences, as growth factor inhibitors. The ability of oligosaccharides of homogeneous composition (Figure 3) but variable length (Figure 4) to inhibit bFGF-induced mitogenesis and motility were examined, and the data suggest that oligosaccharides of less than ten saccharide residues inhibit the biological activity of bFGF. Intact heparin (Figure 2) and dodecasaccharides did not inhibit bFGF-induced mitogenesis (Figures 2 and 5A) or motility (Figure 6), and chondroitin sulphate hexasaccharides and octasaccharides did not inhibit bFGF-induced mitogenesis, suggesting that there is a specific inhibitory action of these oligosaccharides. Samples of 100 µg ml$^{-1}$ hexasaccharides, octasaccharides and decasaccharides inhibited both bFGF-induced mitogenesis and motility, whereas the same concentration of dodecasaccharide (dp12) did not affect either parameter.

© Cancer Research Campaign 1997

It is unlikely that the difference in potency is caused by different molar concentrations of drug, as the octasaccharides were more potent inhibitors than the hexasaccharides at the same concentration (μg $ml^{-1}$), that is, at a lower molar concentration. Although both the deca- and hexasaccharides inhibited the mitogenic activity of bFGF at 100 μg $ml^{-1}$, only the octasaccharides were active at 10 μg $ml^{-1}$, a concentration close to that achieved in patients treated with low-molecular weight heparin (Bara et al, 1985).

The implication of these data is that the length of a heparan sulphate oligosaccharide is a critical determinant of its ability to inhibit growth factor activity and that octasaccharides are very effective by comparison with dp6 and dp10 oligosaccharides. In addition, the data show that the growth factor-activating potential of cell-surface and extracellular matrix heparan sulphate can be overcome by oligosaccharides and that short oligosaccharides are capable of inhibiting growth factor-induced multiplication and motility, processes involved in the progression of cancer.

The results have implications for the biological activity of commercially available low-molecular weight heparins (LMWHs). These contain a spectrum of oligosaccharides that range between tetrasaccharides and hexadecasaccharides (Hirsh and Levine, 1992). Therefore, the LMWHs are likely to contain certain heparin species that support the biological activity of bFGF, while others would inhibit its activity. This suggests that clinical studies of LMWH, as anti-growth factor agents, would be more likely to succeed, if dodecasaccharides and larger oligosaccharides were removed from LMWH, a task that is readily achieved by gel filtration.

The mechanism by which heparan sulphate activates bFGF is unknown, although there are data to suggest that it may act by inducing conformational changes in bFGF (Prestrelski et al, 1992). Other data have shown that two bFGF molecules can be bound by HS, establishing a dimer that is required for growth factor receptor activation (Ornitz et al, 1992). In addition, recent publications have suggested that HS acts as a template by binding to the FGF receptor as well as to bFGF, thereby bringing bFGF into proximity with the FGF receptor (Kan et al, 1993). An oligosaccharide could interfere with any of these processes by occupying HS binding sites on bFGF, preventing the access of functional HS to the molecule. Although less sulphated oligosaccharides would have greater specificity for a heparin-binding growth factor (Hahnenberger et al, 1993; Maccarana et al, 1993; Ishihara, 1994; Ishihara et al, 1994), from the clinical standpoint bovine lung heparin oligosaccharides are attractive anti-growth factor agents, as their structure encompasses features required for the binding of HGF (Lyon et al, 1994*b*) as well as other members of the FGF family (Guimond et al, 1993; Ishihara, 1994). Thus, an inhibitory oligosaccharide derived from bovine lung heparin may have the capacity to inhibit at least four growth factors (HGF, personal communication from M Lyon, JA Deakin and JT Gallagher) that have been implicated in cancer biology, and we are investigating this.

The majority of data (above and Aviezer et al, 1994) suggest that tetrasaccharides and disaccharides have a weak inhibitory effect on the bFGF–FGF receptor interaction and that the optimum length for bFGF inhibitors is an octasaccharide or hexasaccharide. In contrast, Ornitz et al (1995) recently reported that certain non-sulphated heparan disaccharides had the potential to activate bFGF. The physiological importance of this observation is unclear, since these disaccharides would be found only inside degradative lysosomes, where they would not have access to the bFGF–FGF receptor complex. In addition, the data did not show any clear structure–function relationship.

There are a number of heparan sulphate-dependent growth factors, including other FGFs, VEGF, HGF and HB-EGF. The model investigated here serves as a prototype for oligosaccharides that can act as growth factor inhibitors, and since these growth factors have been implicated in both cancer cell growth and angiogenesis (Mustonen and Alitalo, 1995), heparan sulphate and heparin oligosaccharides hold promise as novel anti-cancer agents.

### ABBREVIATIONS

bFGF, basic fibroblast growth factor; CS, chondroitin sulphate; D+T, DMEM, pyruvate, glutamine, penicillin, streptomycin and 10 μg $ml^{-1}$ transferrin; DMEM, Dulbecco's modified Eagle medium; dp, degree of polymerization; dp6, hexasaccharides; EGF, epidermal growth factor; GAG, glycosaminoglycans; GlcNS, *N*-sulphated glucosamine; GlnNS(6S), *N*-sulphated glucosamine 6-*O*-sulphate; HB-EGF, heparin-binding EGF; HGF, hepatocyte growth factor; HPLC, high-performance liquid chromatography; HS, heparan sulphate; IdoA(2S), iduronic acid 2-*O*-sulphate; LMWH, low-molecular weight heparin; PAPS, phosphoadenosine phosphosulphate; VEGF, vascular endothelial growth factor.

### ACKNOWLEDGEMENTS

We would like to thank Merlyn Wellington and Gail Lebens for excellent technical assistance.

### REFERENCES

Aviezer D, Levy E, Safran C, Svahn C, Buddecke E, Schmidt A, David G, Vlodavsky I and Yayon A (1994) Differential structural requirements of heparin and heparan sulphate proteoglycans that promote binding of basic fibroblast growth factor to its receptor. *J Biol Chem* **269**: 114–121

Association of Cancer Physicians (1994) *Review of the Pattern of Cancer Services in England and Wales*. Association of Cancer Physicians: London

Baeuerle PA and Huttner WB (1986) Chlorate – a potent inhibitor of protein sulphation in intact cells. *Biochem Biophys Res Commun* **141**: 870–877

Bara L, Billaud E, Gramond G, Kher A and Savana M (1985) Comparative pharmacokinetics of a low molecular weight heparin (PK10169) and unfractionated heparin after intravenous and subcutaneous administration. *Thromb Res* **39**: 631–636

Coltrini D, Rusnati M, Zoppeti G, Oreste P, Grazioli G, Naggi A and Presta M (1994) Differential effects of mucosal, bovine lung and chemically modified heparin on selected biological properties of basic fibroblast growth factor. *Biochem J* **303**: 583–590

Eisenberger MA, Reyno LM, Jodrell DI, Sinibaldi VJ, Tkaczuk KH, Sridhara R, Zuhowski EG, Lowitt MH, Jacobs SC and Egorin MJ (1993) Suramin, an active drug for prostate cancer: interim observations in a phase I trial. *J Natl Cancer Inst* **85**: 611–621

Gitay-Goren H, Soker S, Vlodavsky I and Neufeld G (1992) The binding of vascular endothelial growth factor to its receptors is dependent on cell surface associated heparin like molecules. *J Biol Chem* **267**: 6093–6098

Guimond S, Maccarana M, Olwin BB, Lindahl U and Rapraeger AC (1993) Activating and inhibitory heparan sequences for FGF-2 (basic FGF). *J Biol Chem* **268**: 23906–23914

Hahnenburger R, Jakobsen AM, Ansari A, Wehler T, Svahn CM and Lindahl U (1993) Low-sulphated oligosaccharides derived from heparan sulphate inhibit normal angiogenesis. *Glycobiology* **3**: 567–573

Higashiyama S, Abraham JA and Klagsbrun M (1993) Heparin-binding EFG-like growth factor stimulation of smooth muscle cell migration: dependence on interactions with cell surface heparan sulphate. *J Cell Biol* **122**: 933–940

Hirsh J and Levine MN (1992) Low molecular weight heparin. *Blood* **29**: 1–17

Ishihara M (1994) Structural requirements in heparin for binding and activation of FGF-1 and FGF-4 are different from that for FGF-2. *Glycobiology* **4**: 817–824

Ishihara M, Tyrell DJ, Stauber GB, Brown S, Cousens LS and Stack RJ (1993) Preparation of affinity fractionated, heparin-derived oligosaccharides and their effects on selected biological activities mediated by basic fibroblast growth factor. *J Biol Chem* **268**: 4675–4683

© Cancer Research Campaign 1997

Ishihara M, Shaklee PN, Yang Z, Liang W, Wei Z, Stack RJ and Holme K (1994) Structural features in heparin which modulate specific biological activities by basic fibroblast growth factor. *Glycobiology* **4**: 451–458

Jayson GC, Evans GS, Pemberton PW, Lobley RW and Allen T (1994) Basic fibroblast growth factor increases the multiplication and migration of a serum free derivative of Caco-2 but does not affect differentiation. *Cancer Res* **54**: 5718–5723

Kan M, Wang F, Xu J, Crabb JW, Hon J and McKeehan WI (1993) An essential heparin-binding domain in the fibroblast growth factor receptor kinase. *Science* **259**: 1918–1921

Lyon M, Deakin JA and Gallagher JT (1994*a*) Liver heparan sulphate structure. *J Biol Chem* **269**: 11208–11215

Lyon M, Deakin JA, Mizuno K, Nakamura T and Gallagher JT (1994*b*) Interaction of hepatocyte growth factor with heparan sulphate. *J Biol Chem* **269**: 11216–11223

Maccarana M, Casu B and Lindahl U (1993) Minimal sequence in heparin/heparan sulphate required for binding of basic fibroblast growth factor. *J Biol Chem* **268**: 23898–23905

Mustonen T and Alitalo K (1995) Endothelial receptor tyrosine kinases involved in angiogenesis. *J Cell Biol* **129**: 895–898

Myers C, Cooper M, Stein C, Larocca R, Walther MM, Weiss G, Choyke P, Dawson N, Steinberg S, Uhrich MM, Cassidy J, Kohler DR, Trepel J and Linehan WM (1992) Suramin: a novel growth factor antagonist with activity in hormone refractory prostate cancer. *J Clin Oncol* **10**: 881–889

Olwin B and Rapraeger A (1992) Repression of myogenic differentiation by aFGF, bFGF and K-FGF is dependent on cellular heparan sulphate. *J Cell Biol* **118**: 631–639

Ornitz DM, Yayon A, Flanagan JG, Svahn CM, Levi E and Leder P (1992) Heparin is required for cell free binding of basic fibroblast growth factor to a soluble receptor and for mitogenesis in whole cells. *Mol Cell Biol* **12**: 240–247

Ornitz DM, Herr AB, Nilsson M, Westman J, Svahn CM and Waksman G (1995) FGF binding and FGF receptor activation by synthetic heparin-derived di- and trisaccharides. *Science* **268**: 432–436

Prestrelski SJ, Fox GM and Arakawa T (1992) Binding of heparin to basic fibroblast growth factor induces a conformational change. *Arch Biochem Biophys* **293**: 314–319

Rapraeger A, Krufka A and Olwin BB (1991) Requirement of heparan sulphate for bFGF mediated fibroblast growth and myoblast differentiation. *Science* **252**: 1705–1707

Shively JE and Conrad HE (1976) Formation of anhydrosugars in the chemical depolymerisation of heparin. *Biochemistry* **15**: 3932–3942

Turnbull JE and Gallagher JT (1991) Distribution of iduronate 2-sulphate residues in heparan sulphate. Evidence for an ordered polymeric structure. *Biochem J* **273**: 553–559

Turnbull JE, Fernig DG, Ke Y, Wilkinson MC and Gallagher JT (1992) Identification of the basic fibroblast factor binding sequence in fibroblast heparan sulphate. *J Biol Chem* **267**: 10337–10341

Walker A, Turnbull JE and Gallagher JT (1994) Specific heparan sulphate saccharides mediate the activity of basic fibroblast growth factor. *J Biol Chem* **269**: 931–935

Yayon A, Klagsbrun M, Esko JD, Leder P and Ornitz DM (1991) Cell surface, heparin-like molecules are required for binding of basic fibroblast growth factor to its high affinity receptor. *Cell* **64**: 841–848

 © Cancer Research Campaign 1997


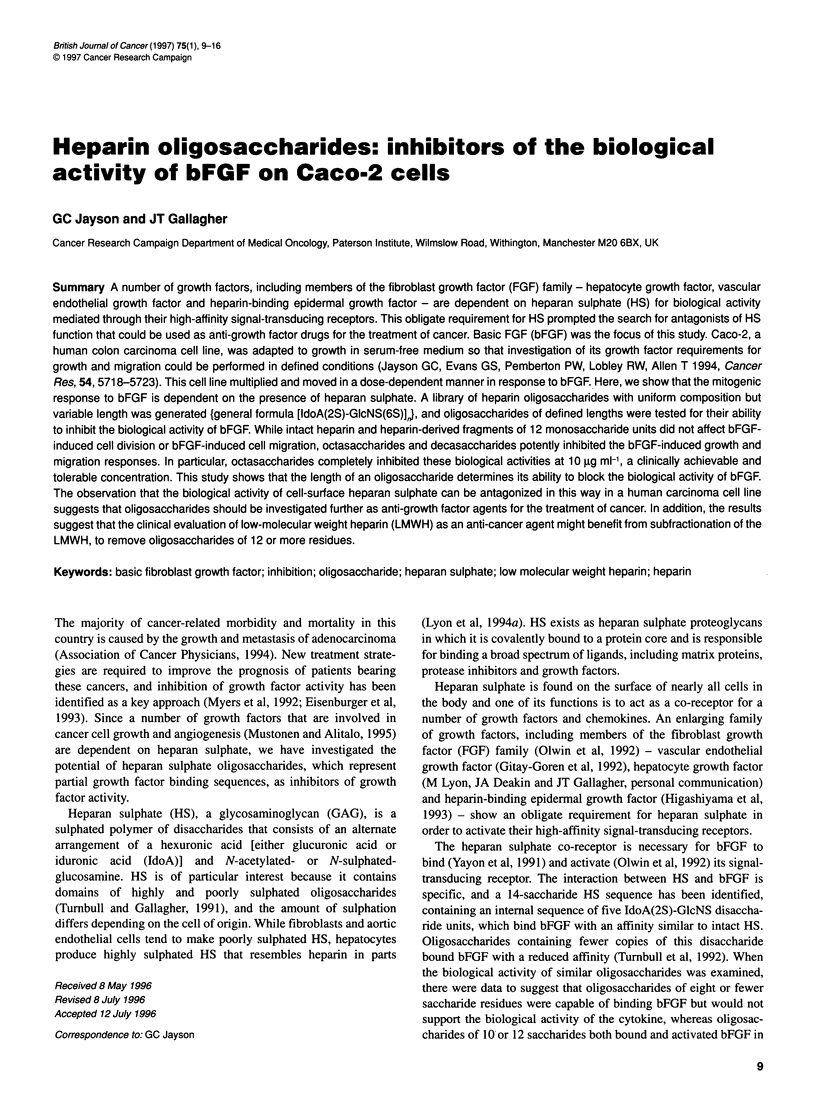

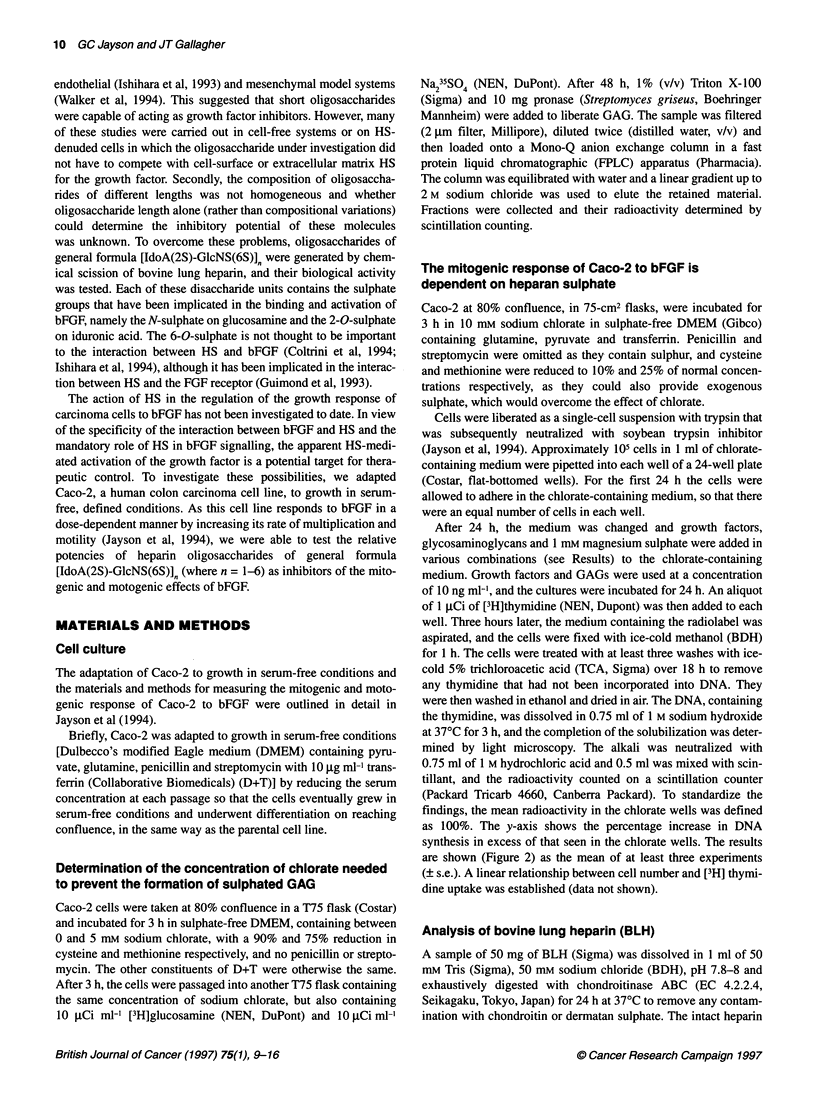

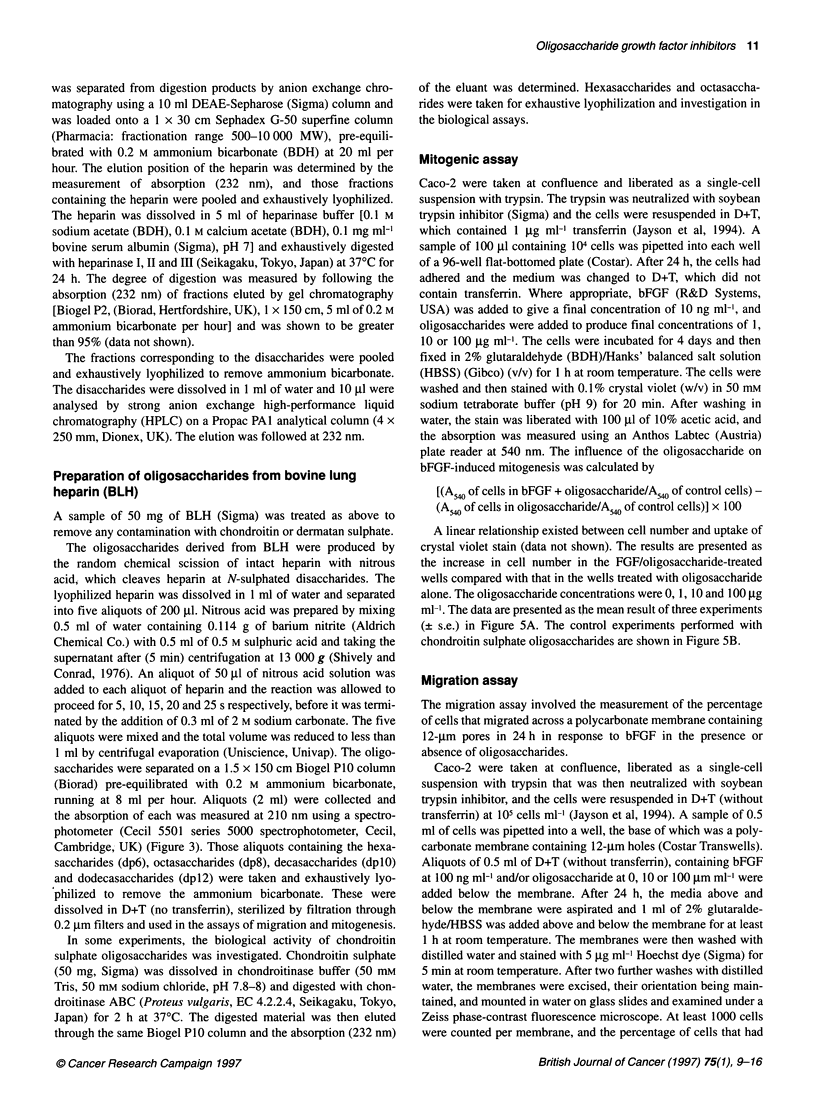

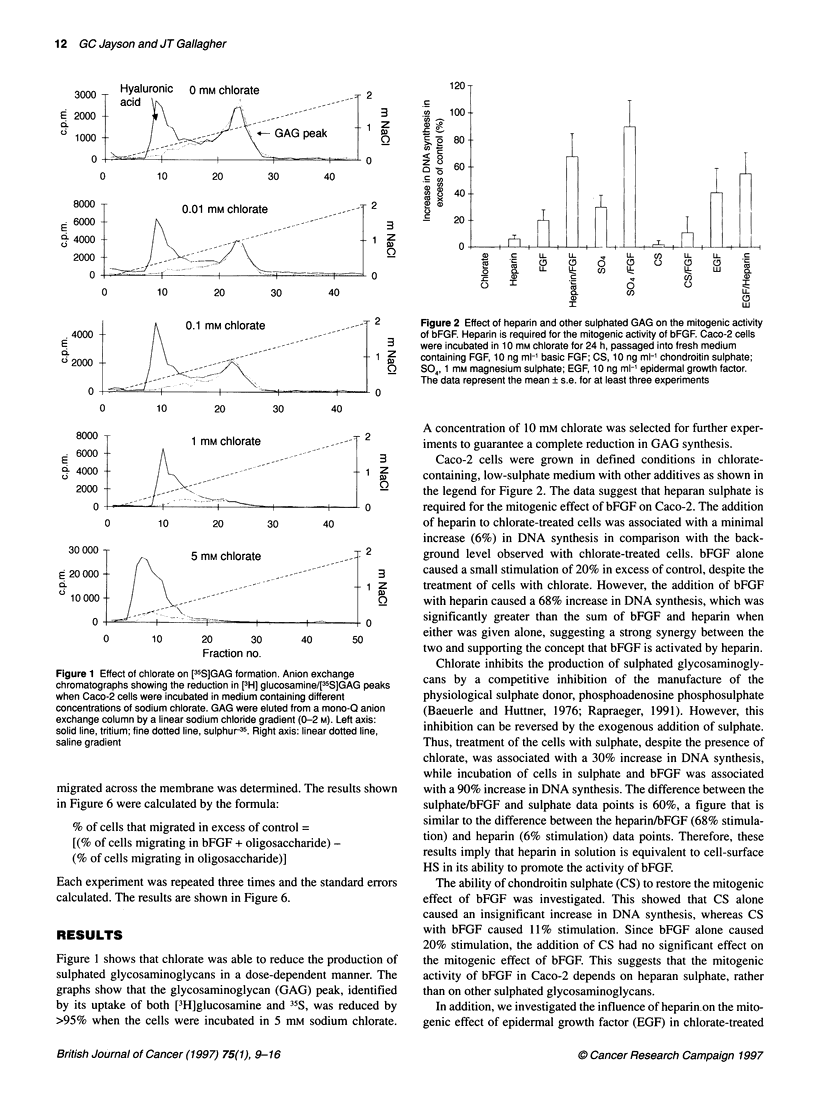

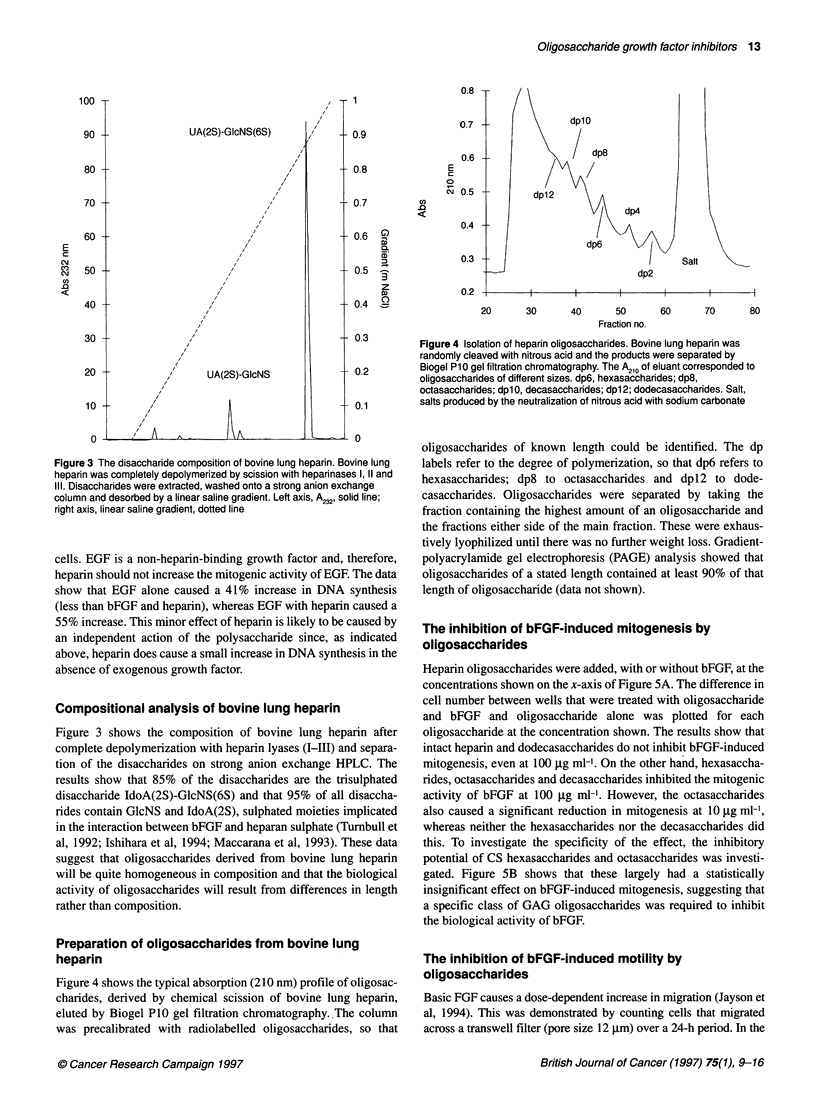

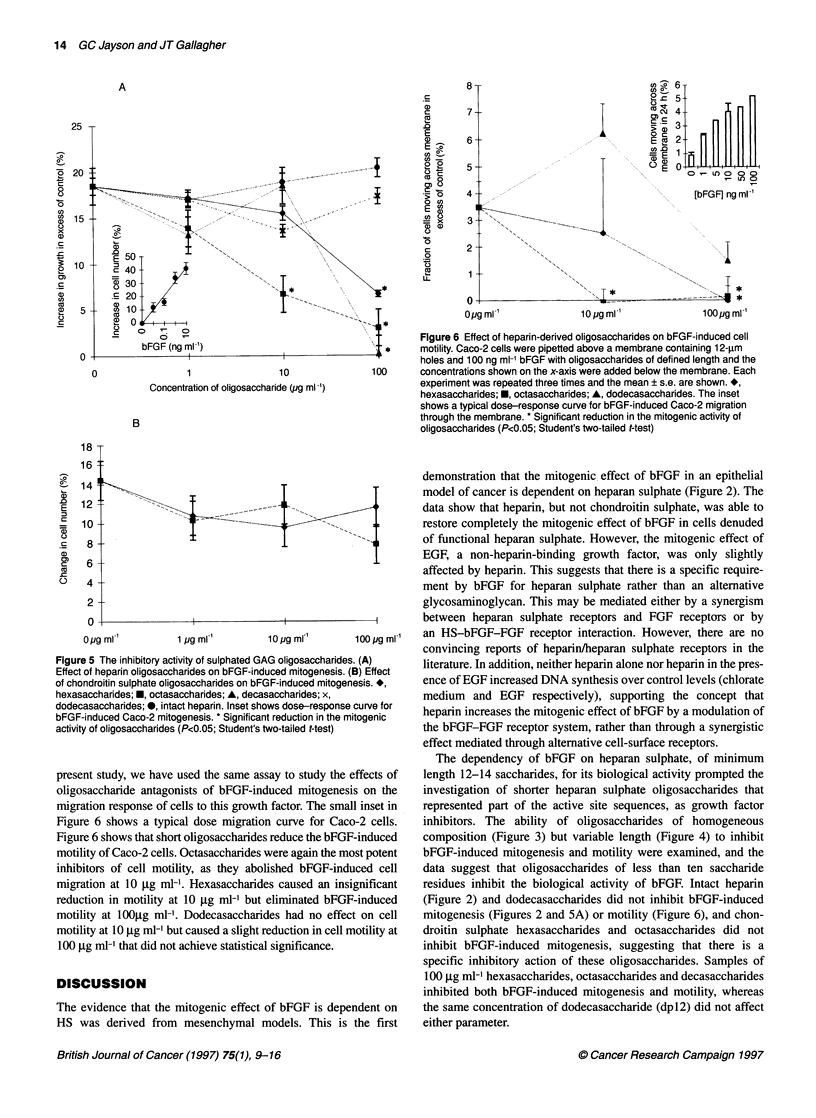

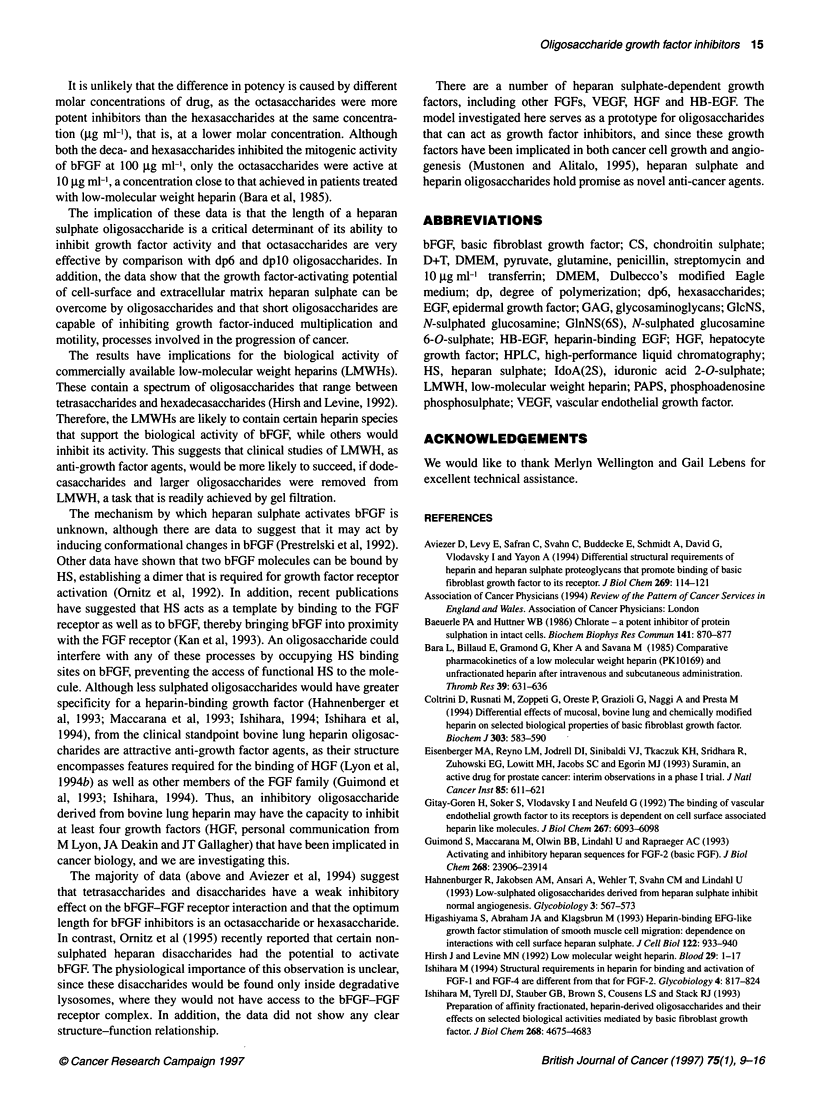

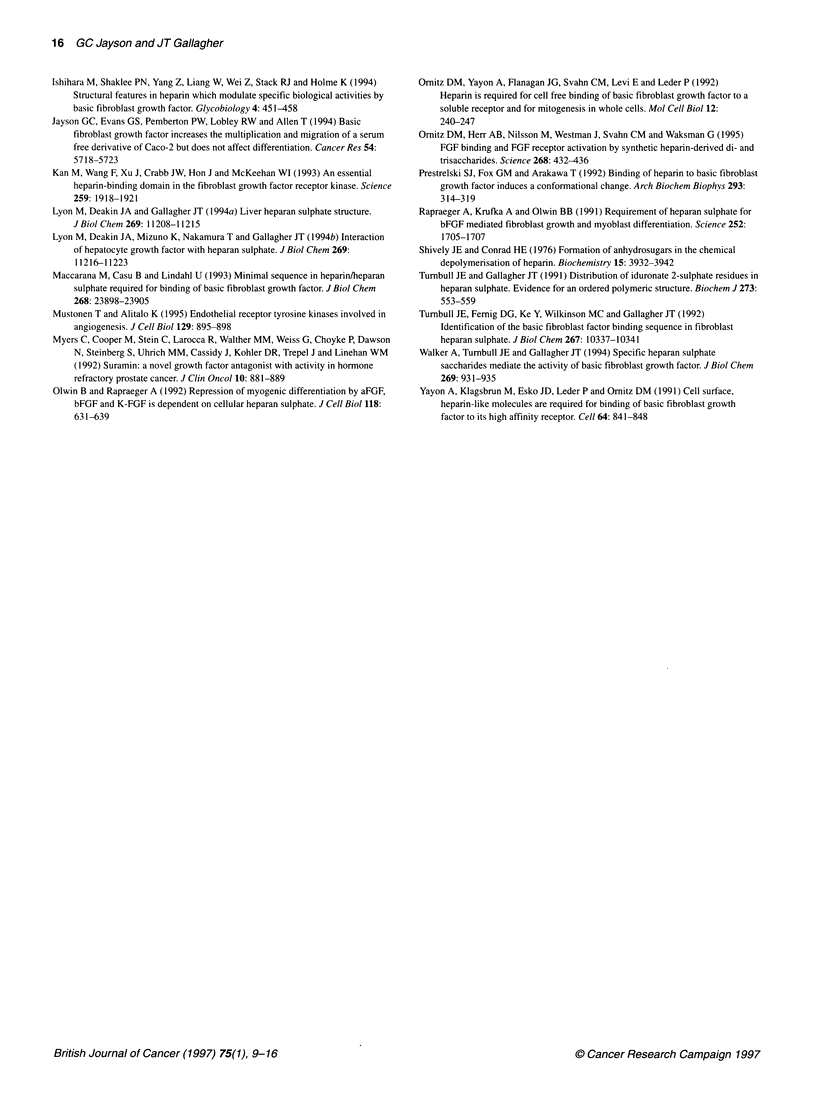


## References

[OCR_01051] Aviezer D., Levy E., Safran M., Svahn C., Buddecke E., Schmidt A., David G., Vlodavsky I., Yayon A. (1994). Differential structural requirements of heparin and heparan sulfate proteoglycans that promote binding of basic fibroblast growth factor to its receptor.. J Biol Chem.

[OCR_01062] Baeuerle P. A., Huttner W. B. (1986). Chlorate--a potent inhibitor of protein sulfation in intact cells.. Biochem Biophys Res Commun.

[OCR_01065] Bara L., Billaud E., Gramond G., Kher A., Samama M. (1985). Comparative pharmacokinetics of a low molecular weight heparin (PK 10 169) and unfractionated heparin after intravenous and subcutaneous administration.. Thromb Res.

[OCR_01072] Coltrini D., Rusnati M., Zoppetti G., Oreste P., Grazioli G., Naggi A., Presta M. (1994). Different effects of mucosal, bovine lung and chemically modified heparin on selected biological properties of basic fibroblast growth factor.. Biochem J.

[OCR_01078] Eisenberger M. A., Reyno L. M., Jodrell D. I., Sinibaldi V. J., Tkaczuk K. H., Sridhara R., Zuhowski E. G., Lowitt M. H., Jacobs S. C., Egorin M. J. (1993). Suramin, an active drug for prostate cancer: interim observations in a phase I trial.. J Natl Cancer Inst.

[OCR_01085] Gitay-Goren H., Soker S., Vlodavsky I., Neufeld G. (1992). The binding of vascular endothelial growth factor to its receptors is dependent on cell surface-associated heparin-like molecules.. J Biol Chem.

[OCR_01090] Guimond S., Maccarana M., Olwin B. B., Lindahl U., Rapraeger A. C. (1993). Activating and inhibitory heparin sequences for FGF-2 (basic FGF). Distinct requirements for FGF-1, FGF-2, and FGF-4.. J Biol Chem.

[OCR_01095] Hahnenberger R., Jakobson A. M., Ansari A., Wehler T., Svahn C. M., Lindahl U. (1993). Low-sulphated oligosaccharides derived from heparan sulphate inhibit normal angiogenesis.. Glycobiology.

[OCR_01100] Higashiyama S., Abraham J. A., Klagsbrun M. (1993). Heparin-binding EGF-like growth factor stimulation of smooth muscle cell migration: dependence on interactions with cell surface heparan sulfate.. J Cell Biol.

[OCR_01104] Hirsh J., Levine M. N. (1992). Low molecular weight heparin.. Blood.

[OCR_01119] Ishihara M., Shaklee P. N., Yang Z., Liang W., Wei Z., Stack R. J., Holme K. (1994). Structural features in heparin which modulate specific biological activities mediated by basic fibroblast growth factor.. Glycobiology.

[OCR_01106] Ishihara M. (1994). Structural requirements in heparin for binding and activation of FGF-1 and FGF-4 are different from that for FGF-2.. Glycobiology.

[OCR_01109] Ishihara M., Tyrrell D. J., Stauber G. B., Brown S., Cousens L. S., Stack R. J. (1993). Preparation of affinity-fractionated, heparin-derived oligosaccharides and their effects on selected biological activities mediated by basic fibroblast growth factor.. J Biol Chem.

[OCR_01124] Jayson G. C., Evans G. S., Pemberton P. W., Lobley R. W., Allen T. (1994). Basic fibroblast growth factor increases the multiplication and migration of a serum-free derivative of CACO-2 but does not affect differentiation.. Cancer Res.

[OCR_01130] Kan M., Wang F., Xu J., Crabb J. W., Hou J., McKeehan W. L. (1993). An essential heparin-binding domain in the fibroblast growth factor receptor kinase.. Science.

[OCR_01144] Maccarana M., Casu B., Lindahl U. (1993). Minimal sequence in heparin/heparan sulfate required for binding of basic fibroblast growth factor.. J Biol Chem.

[OCR_01149] Mustonen T., Alitalo K. (1995). Endothelial receptor tyrosine kinases involved in angiogenesis.. J Cell Biol.

[OCR_01153] Myers C., Cooper M., Stein C., LaRocca R., Walther M. M., Weiss G., Choyke P., Dawson N., Steinberg S., Uhrich M. M. (1992). Suramin: a novel growth factor antagonist with activity in hormone-refractory metastatic prostate cancer.. J Clin Oncol.

[OCR_01159] Olwin B. B., Rapraeger A. (1992). Repression of myogenic differentiation by aFGF, bFGF, and K-FGF is dependent on cellular heparan sulfate.. J Cell Biol.

[OCR_01170] Ornitz D. M., Herr A. B., Nilsson M., Westman J., Svahn C. M., Waksman G. (1995). FGF binding and FGF receptor activation by synthetic heparan-derived di- and trisaccharides.. Science.

[OCR_01164] Ornitz D. M., Yayon A., Flanagan J. G., Svahn C. M., Levi E., Leder P. (1992). Heparin is required for cell-free binding of basic fibroblast growth factor to a soluble receptor and for mitogenesis in whole cells.. Mol Cell Biol.

[OCR_01175] Prestrelski S. J., Fox G. M., Arakawa T. (1992). Binding of heparin to basic fibroblast growth factor induces a conformational change.. Arch Biochem Biophys.

[OCR_01180] Rapraeger A. C., Krufka A., Olwin B. B. (1991). Requirement of heparan sulfate for bFGF-mediated fibroblast growth and myoblast differentiation.. Science.

[OCR_01185] Shively J. E., Conrad H. E. (1976). Formation of anhydrosugars in the chemical depolymerization of heparin.. Biochemistry.

[OCR_01194] Turnbull J. E., Fernig D. G., Ke Y., Wilkinson M. C., Gallagher J. T. (1992). Identification of the basic fibroblast growth factor binding sequence in fibroblast heparan sulfate.. J Biol Chem.

[OCR_01189] Turnbull J. E., Gallagher J. T. (1991). Distribution of iduronate 2-sulphate residues in heparan sulphate. Evidence for an ordered polymeric structure.. Biochem J.

[OCR_01199] Walker A., Turnbull J. E., Gallagher J. T. (1994). Specific heparan sulfate saccharides mediate the activity of basic fibroblast growth factor.. J Biol Chem.

[OCR_01204] Yayon A., Klagsbrun M., Esko J. D., Leder P., Ornitz D. M. (1991). Cell surface, heparin-like molecules are required for binding of basic fibroblast growth factor to its high affinity receptor.. Cell.

